# Dapagliflozin alleviates myocardial ischemia/reperfusion injury by reducing ferroptosis *via* MAPK signaling inhibition

**DOI:** 10.3389/fphar.2023.1078205

**Published:** 2023-02-20

**Authors:** Weixiang Chen, Yue Zhang, Zuoxiang Wang, Mingyue Tan, Jia Lin, Xiaodong Qian, Hongxia Li, Tingbo Jiang

**Affiliations:** Department of Cardiology, The First Affiliated Hospital of Soochow University, Suzhou, China

**Keywords:** myocardial ischemia/reperfusion, dapagliflozin, ferroptosis, MAPK pathway, treatment

## Abstract

Reperfusion is essential for ischemic myocardium but paradoxically leads to myocardial damage that worsens cardiac functions. Ferroptosis often occurs in cardiomyocytes during ischemia/reperfusion (I/R). The SGLT2 inhibitor dapagliflozin (DAPA) exerts cardioprotective effects independent of hypoglycemia. Here, we investigated the effect and potential mechanism of DAPA against myocardial ischemia/reperfusion injury (MIRI)-related ferroptosis using the MIRI rat model and hypoxia/reoxygenation (H/R)-induced H9C2 cardiomyocytes. Our results show that DAPA significantly ameliorated myocardial injury, reperfusion arrhythmia, and cardiac function, as evidenced by alleviated ST-segment elevation, ameliorated cardiac injury biomarkers including cTnT and BNP and pathological features, prevented H/R-triggered cell viability loss *in vitro*. *In vitro* and *in vivo* experiments showed that DAPA inhibited ferroptosis by upregulating the SLC7A11/GPX4 axis and FTH and inhibiting ACSL4. DAPA notably mitigated oxidative stress, lipid peroxidation, ferrous iron overload, and reduced ferroptosis. Subsequently, network pharmacology and bioinformatics analysis suggested that the MAPK signaling pathway was a potential target of DAPA and a common mechanism of MIRI and ferroptosis. DAPA treatment significantly reduced MAPK phosphorylation *in vitro* and *in vivo*, suggesting that DAPA might protect against MIRI by reducing ferroptosis through the MAPK signaling pathway.

## 1 Introduction

Myocardial infarction (MI) is one of the leading causes of death worldwide (Li S. et al., 2022; Asaria et al., 2022). Clinically, timely reperfusion is essential to rescue ischemic myocardial tissue by reducing infarct size, maintaining left ventricular systolic function, and preventing the occurrence of heart failure. However, the treatment itself may paradoxically induce myocardial injury and worsen cardiac function; this phenomenon is known as myocardial ischemia-reperfusion injury (MIRI) (Yellon and Hausenloy, 2007). Existing studies have shown that reperfusion injury accounts for 50% of the total myocardial infarction area (Hausenloy and Yellon, 2015). Mechanisms underlying MIRI are complicated and include apoptosis, pyroptosis, necroptosis, autophagy, energy metabolism disorder, intracellular calcium overload, cell inflammation, and oxidative stress (He J. et al., 2022). Multiple strategies for alleviating ischemic myocardial infarction and dysfunction have had limited effects in animal models and clinical applications, suggesting that other mechanisms need to be explored.

Ferroptosis remains a novel type of programmed cell death (PCD) characterized by intracellular iron overload, glutathione depletion, and lipid peroxidation (Dixon et al., 2012; Fang et al., 2022) and has been involved in the pathogenesis of various cardiovascular diseases, including MIRI (Ju et al., 2021). During the MIRI period, the accumulation of iron leads to the overgeneration of reactive oxygen species ROS, resulting in lipid peroxidation and ferroptosis. Therefore, selective reduction of ferroptosis is a promising strategy for alleviating myocardial injury. Although many studies have explored the use of drugs or nanoparticles to inhibit ferroptosis to protect against MIRI (Zhang et al., 2021; Lin J. H et al., 2022), direct clinical use is not yet available. Therefore, it is a shortcut to explore drugs that can inhibit ferroptosis among the medicines already in clinical application to achieve the goal of alleviating I/R injury.

Dapagliflozin (DAPA), a sodium-glucose sodium-glucose cotransporter subtype 2 inhibitor (SGLT2i), is a class of antidiabetic drugs that reduces blood glucose levels by increasing glucose and natriuresis and is used in the treatment of type 2 diabetes mellitus (T2DM) (Vallon, 2015; Verma and McMurray, 2018; Cowie and Fisher, 2020; Scheen, 2020; Silva Dos Santos et al., 2020). Recent clinical trials of DAPA-HF (McMurray et al., 2019) expand the use of SGLT2i not only in heart failure patients with T2DM but also in patients without T2DM, showing the advantage of DAPA in the primary cardiovascular outcome (cardiovascular death or hospitalization). The cardiovascular benefits of SGLT2i in clinical application prompted researchers to further explore its possible cardioprotective mechanism. DAPA showed a 1200-fold selectivity for SGLT2 over SGLT1 (Zugner et al., 2022), and the effects of DAPA on mitochondrial complex I activity and amino acid metabolites have been reported (Uthman et al., 2019; Mulder et al., 2020). Animal studies have suggested that DAPA may protect the myocardium from myocardial infarction (Yu et al., 2021), obesity-related cardiomyopathy (Lin K. et al., 2022), and methamphetamine-induced cardiomyopathy (He S. et al., 2022). Cardiomyocytes and cardiac tissues lack SGLT2 expression, and the cardioprotective effects of SGLT2 inhibitors should be mediated by other targets and signaling pathways. Hence, discussions of the mechanism of SGLT2i have shown that it can inhibit myocardial inflammation (Lin K. et al., 2022), reduce oxidative stress (Nikolaou et al., 2021), regulate autophagy (Yu et al., 2021), improve mitochondrial function, and reduce myocardial cell apoptosis (Nikolaou et al., 2022). However, the MIRI-improving effects of DAPA by inhibiting cardiomyocyte ferroptosis have not been evaluated. The objective of the present study was to investigate whether DAPA could attenuate I/R-induced ferroptosis in cardiomyocytes and the underlying mechanisms.

## 2 Materials and methods

### 2.1 Chemicals and reagents

Dapagliflozin was obtained from MCE (Cat. HY-10450). Ferrostatin-1 (Fer-1) was purchased from Selleck Chemicals (Cat# S7243). Drugs were separately dissolved in DMSO and added to the culture medium 12 h before H/R. For *in vivo* experiments, DAPA was purchased from commercially available dapagliflozin tablets (Forxiga Tab 10 mg, AstraZeneca AB, United States). DAPA was diluted with sterile water for injection (WFI) and given to animals by intragastric administration, as previously shown (Nikolaou et al., 2022). Primary antibodies against β-tubulin (1:5,000, Cat. AC021), GPX4 (1:1,000, A1933), SLC7A11 (1:1,000,A2413), ACSL4 (1:1,000, A6826), PTGS2 (1:1,000, A1253), and FTH1 (1:1,000, A19544) were purchased from ABclonal (Wuhan, China); FTMT (1:200,aa65-227) was purchased from LifeSpan BioSciences (Seattle, WA, United States); p-ERK (1:1,000,#4370), ERK (1:1,000, #4695), p-P38 (1:1,000, #4511), and P38 (1:1,000, #8690) were purchased from Cell Signaling Technology (Beverly, MA, United States); p-JNK (1:1,000, ab76572)), and JNK (1:1,000, ab208035) were purchased from Abcam (San Francisco, CA).

### 2.2 Cell culture and treatment

H9C2, the rat cardiomyocyte cell line, was purchased from the Shanghai Institute of Cell Biology, Chinese Academy of Science (Shanghai, China). The cells were cultured with Dulbecco’s modified Eagle’s medium (DMEM) containing 10% fetal bovine serum (FBS), 100 U/mL penicillin, and 100 mg/mL streptomycin (Beyotime, Shanghai, China) at 37°C with 5% CO_2_. The cells were treated with the H/R model as reported previously (Son et al., 2020). H9C2 cardiomyocytes were replaced with FBS-free and glucose-free DMEM and exposed to a hypoxic environment of 95% N_2_ and 5% CO_2_ at 37°C for 4 h, while the control groups were cultured under normal conditions. H9C2 cells were then replaced with ordinary medium and placed in an ordinary incubator for 2 h to simulate reoxygenation injury. Two concentrations of DAPA (2.5 or 5 μM)were preadministered for 12 h.

### 2.3 Detection of cell viability and LDH

Cell viability was measured using a Cell Counting Kit-8 (CCK-8) (CK04, Dojindo). After treatment with H/R on a 96-well plate, 10 μL CCK-8 solution was added and incubated at 37°C for 1 h. Then, the OD of each well was measured at 450 nm using a microplate reader (Thermo Fisher Scientific, United States of America). A cytotoxicity lactate dehydrogenase (LDH) Assay Kit-WST (CK12, Dojindo) was used to detect the release of lactate dehydrogenase to further detect the degree of cell damage. The reagent was added according to the product instructions, and the absorbance at 490 nm was measured immediately to calculate the cell damage rate.

### 2.4 Determination of GSH, SOD, MDA, and iron levels

H9C2 cardiomyocytes were washed twice with ice-cold PBS and then lysed. The glutathione (GSH) content was measured using a GSH and GSSG assay kit (S0053, Beyotime), and the optical density was measured at 405 nm. A lipid oxidation detection kit (S0131S, Beyotime) was used to detect the level of malondialdehyde (MDA), and the optical density value was measured at 535 nm. SOD enzyme activity was detected by a total SOD activity detection kit (WST-8 method) (S0101S, Beyotime), and the optical density value was measured at 450 nm. The Fe^2+^ concentrations of cardiac tissue and cell samples were determined using a Ferrous Iron Colorimetric Assay Kit (E-BC-K773-M, Elabscience, Wuhan, China), measuring absorbance at 590 nm using a microplate reader.

### 2.5 Measurement of intracellular ROS

Intracellular ROS were detected by fluorescence 2′,7′-dichlorofluorescein diacetate (DCFH-DA) (S0033M, Beyotime, Shanghai, China) and Dihydroethidium (S0063, Beyotime). The cells were incubated with 10 μM DCFH-DA or 5 μM Dihydroethidium diluted with FBS-free DMEM for 20 min at 37°C and then washed twice with FBS-free medium. Fluorescence at an excitation wavelength of 488 nm was measured by fluorescence microscopy or flow cytometry.

### 2.6 Flow cytometry-based intracellular lipid peroxidation accumulation

Cells were seeded in 6-well plates and pretreated with dapagliflozin as indicated. After H/R, the cells were incubated with 10 μM C11 BODIPY 581/591 (RM02821, ABclonal) for 1 h at 37°C in the dark and then analyzed using a flow cytometer. BODIPY emission was detected using the FL one channel. As a conservative estimate, 10,000 cells were collected for data analysis.

### 2.7 Detection of mitochondrial membrane potential (ΔΨm)

ΔΨm was detected using a JC-1 fluorescent probe (S2003, Beyotime, China), a non-toxic cell-permeable cationic fluorescent dye. Briefly, after treatment, H9C2 cells were incubated with 1 mL JC-1 staining solution in a 37°C incubator for 20 min, washed with JC-1 staining buffer twice, cultured with 1 mL of culture medium according to the experimental protocols, and then removed in an ice bath. Fluorescence was immediately captured with a fluorescence microscope. The JC-1 monomers were excited with a 488 nm helium-neon laser for green fluorescence and imaged through a 525 nm-long path filter. In addition, the JC-1 aggregates were excited with red fluorescence by a 543 nm He-Ne laser line and imaged through a 590 nm-long path filter.

### 2.8 Animals and experimental protocols

Based on “Practical guidelines for Rigor and Repeatability in Preclinical and clinical studies of cardiac protection” (Botker et al., 2018), the study was conducted in adult male SD rats aged 8–12 weeks weighing 250–300 g. The experimental protocols of the animals involved in this study were approved by the Laboratory Animal Research Committee of Soochow University. Rats were kept in pathogen-free, temperature-controlled environments (20°C–25°C) and specific facilities with 12-h light/dark cycles, with a maximum of six per cage and free feeding on conventional laboratory animal feed. The rats were randomly divided into three groups (*n* = 5 per group): Sham group (saline + sham operation), I/R group (saline + I/R); Dapa group (dapagliflozin 10 mg/kg/day + I/R). DAPA or saline was intragastrically administered once daily for 5 days. On the sixth day, the rats underwent myocardial ischemia for 30 min followed by reperfusion for 2 h. Briefly, adult male SD rats were anesthetized with 50 mg/kg sodium pentobarbital and placed in the supine position on a 37°C heating pad. During the experiment, a standard limb II lead electrocardiogram was performed continuously. The tracheal incision was intubated, and mechanical ventilation was connected with a ventilator. After the left thoracic incision, 6-0 silk thread was sutured at the root of the anterior descending branch of the left coronary artery (LAD), positioned 2 mm below the intersection of the left atrial appendage and arterial conus, and a slip-knot was made. After 30 min of ischemia, the knot was released, and reperfusion was performed for 2 h. In the sham group, the same thoracotomy was performed without ligating the coronary arteries. The animals were then sacrificed for subsequent experiments.

### 2.9 Transthoracic echocardiography

Transthoracic echocardiography was performed at baseline (before experiments) and after reperfusion in anesthetized rats using a Visual Sonic Vevo2100 system with an MS-250 transducer. Heart rate and left ventricular (LV) parameters, including diastolic and systolic wall thickness, LV end-diastolic and end-systolic diameters, and LV functional parameters, including left ventricular ejection fraction (LVEF) and LV fractional shortening (LVFS), LV end-systolic interior diameter (LVID.s), end-diastolic interior diameter (LVID. d), were measured from a 2D short axis at the papillary muscle level in M-mode images using Vevo 2,100 software.

### 2.10 Hematoxylin and eosin staining

The heart was removed and fixed with 4% paraformaldehyde overnight, embedded in paraffin, and sliced into 5-μm-thick sections. Left ventricular specimens were stained with hematoxylin and eosin (H&E) according to a previously reported method (Fang et al., 2019) and photographed by light microscopy.

### 2.11 Detection of serum cTnT and BNP levels, and infarcted area measurements

Blood samples were collected after I/R, and serum was obtained after centrifugation at 2000 × g for 20 min. Cardiac troponin T (TNT) and brain natriuretic peptide (BNP) levels were determined using commercial ELISA kits (LunChangshuo Biotech, China) according to the manufacturer’s instructions. Infarct size determination was performed using Evans blue and 2,3,5-triphenyl tetrazolium chloride monohydrate (TTC) (G3005, Solarbio, Beijing, China) double staining. Briefly, following 2 h of reperfusion, the hearts were excised and 2 ml of a 1% solution of Evans Blue dye was retrogradely perfused into the aorta. After perfusion, the heart was frozen at −20°C for 30 min, and sliced into five cross-sections from apex to base. The sections were incubated with 1% TTC at 37°C for 10 min and then fixed in 10% formalin solution for 24 h. The heart sections were analyzed using ImageJ software, the blue area determined the non-ischemic region, and areas were summed and calculations of the White Area (Infarct area, IA)/total red + white area (Area at risk, AAR) for each heart were determined. The myocardial infarction size is a percentage of the area at risk.

### 2.12 Quantitative real-time PCR

Total mRNA was extracted from H9C2 cells or heart homogenates using commercial RNA extraction kits (RC112-01, Vazyme, China). The concentration and purity of the samples were measured using a NanoDrop One spectrophotometer (Thermo Scientific, Waltham, MA). RNA was reverse-transcribed by ABScript III RT Master Mix for qPCR with gDNA Remover (RK20429, ABclonal) according to the manufacturer’s instructions. Quantitative PCR was performed with an AB7500 Fast Real-Time System using Genious 2X SYBR green (RK21204, Abclonal). The sequences of the primers are shown in Supplementary Table S1. The amplification conditions for PCR were as follows (in a total volume of 10 μl): 95°C for 10 min followed by 36 cycles of denaturation at 65°C for 5 min. Cycle threshold (Ct) values were determined by the comparative Ct method and normalized to GAPDH levels.

### 2.13 Western blot and immunofluorescence

For Western blotting, cells or tissues were lysed using RIPA buffer containing 1% protease inhibitor and phosphatase inhibitor for 30 min and then centrifuged at 12000 × g for 15 min at 4°C to collect the supernatant. The protein concentrations were measured using a Barford protein assay kit (P0006C, Beyotime). The samples were heated with a loading buffer at 95°C for 15 min. Approximately 20 µg of protein was separated by 10%–12.5% SDS‒PAGE and then transferred to PVDF membranes. After blocking with 5% BSA for 1 h, the membranes were incubated overnight with a specific primary antibody at 4°C. The immunoreactive bands were incubated with horseradish peroxidase secondary antibody for 1 h and exposed using a gel imager (Bio-Rad, CA, United States). Protein bands were visualized using an ECL kit (FUDE Biological Technology, Hangzhou, China) and quantified using ImageJ analysis software version 1.8.0. The relative quantity of the target protein was normalized to β-tubulin. For immunofluorescence, samples were treated with primary antibody followed by incubation with Alexa Fluor 488-conjugated anti-rabbit IgG (1:200, Ab150117).

### 2.14 Bioinformatics and network pharmacology analysis

#### 2.14.1 Screening of MIRI-related and ferroptosis-related genes and prediction of potential dapagliflozin targets

Ischemia-reperfusion pathogenic targets were collected online from the Mendelian Inheritance in Man (OMIM, http://omim.org) (Amberger et al., 2015) and Gene Cards (http://www.genec ards.org) databases (Stelzer et al., 2016) using the keywords “Myocardial ischemia Reperfusion injury” or “ischemia Reperfusion injury” or “Myocardial ischemia Reperfusion”. We selected genes that scored above the median as potential targets. The structural formula of DAPA was searched in the PubChem database (https://pubchem.ncbi.nlm.nih.gov) (Kim et al., 2021). Target prediction for DAPA employed Swiss targets (http://www. swisstargetprediction. ch) (Daina et al., 2019). Prediction targets with probability >0.1 were selected for subsequent analysis.

#### 2.14.2 Common targets of MIRI, ferroptosis, and dapagliflozin

To determine the dapagliflozin interaction in the treatment of MIRI and ferroptosis, we adopted the online drawing tool (https://hiplotacademic.com) (Li J. et al., 2022) to draw a Venn diagram. The overlap is the potential therapeutic targets of DAPA for MIRI and ferroptosis. PPI networks for these common targets were constructed through the STRING11.5 platform (https://cn.string-db.org/) (Szklarczyk et al., 2019) and were obtained by hiding the disconnected nodes. The plug-in MCODE of Cytoscape 3.9.1 (http://cytos.cape.org/) (Shannon et al., 2003) was used for cluster analysis of the PPI network, and the targets with the highest score of MCODE analysis were selected as the potential hub targets. Gene Ontology (GO) functional analysis and Kyoto Encyclopedia of Genes and Genomes (KEGG) pathway enrichment analysis were performed by the DAVID database (Huang et al., 2009) and KOBAS 3.0 database (Wu et al., 2006).

### 2.15 Statistical analysis

The data in this study represent at least three independent experiments, and the values are expressed as the mean ± standard deviation. Statistical analysis was performed using GraphPad Prism 8.0 software (San Diego, CA, United States). First, the normal distribution was tested, followed by a homogeneity test of variance using Levene’s test. Data from more than two groups were compared using one-way ANOVA followed by Tukey’s correction for *post hoc* multiple comparisons (equal variances) or Welch ANOVA tests followed by Dunnett’s T3 multiple comparisons test (Unequal variances). Two-way ANOVA for repeated measures (cardiac echocardiography parameters) followed by Sidak’s multiple comparison tests. *p* < 0.05 was considered to be statistically significant.

## 3 Results

### 3.1 Dapagliflozin protects H9C2 cardiomyocytes against H/R-induced injury

**FIGURE 1:**
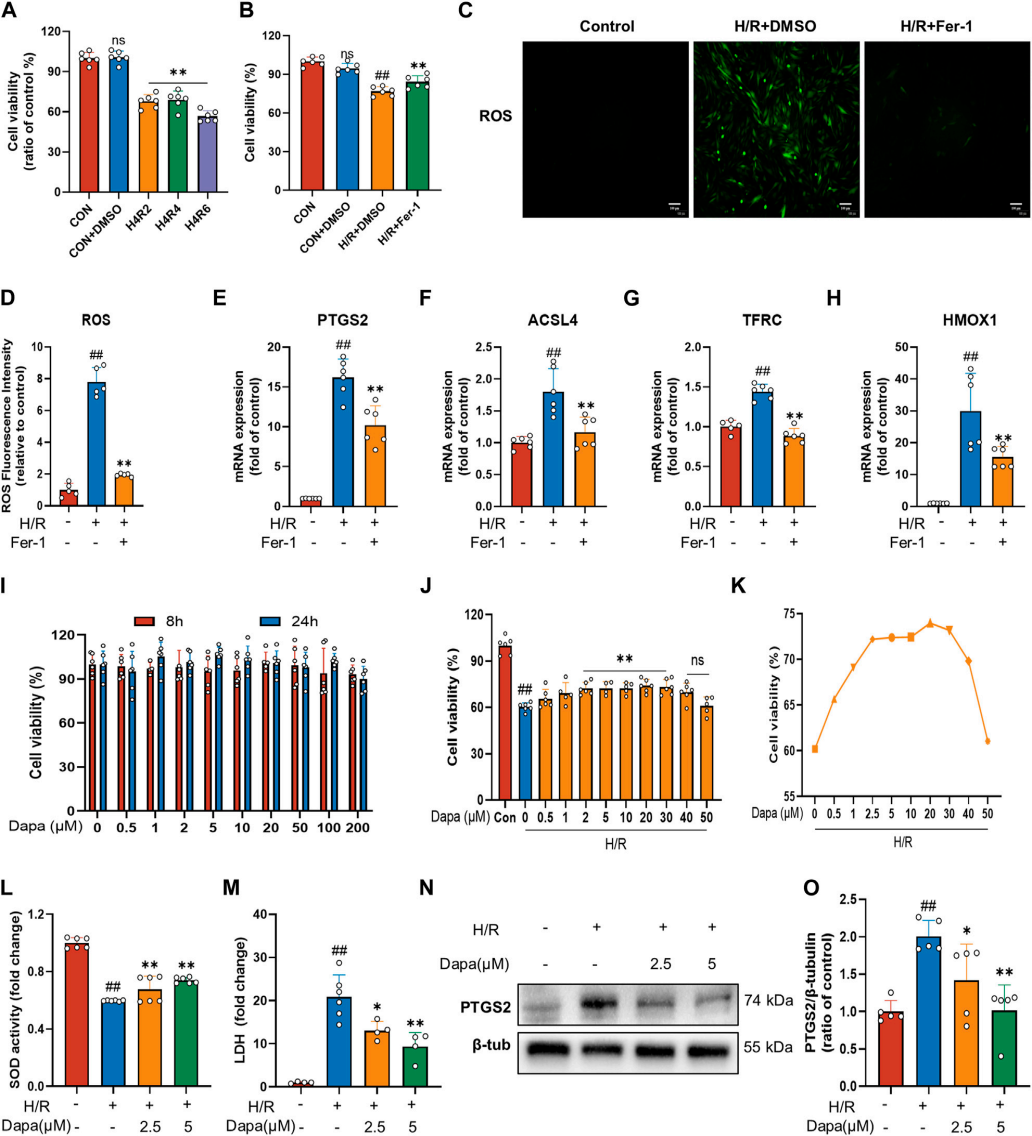
Dapagliflozin protects H9C2 cells against H/R-triggered cell injury. **(A)** Viability of H9C2 cells treated with hypoxia for 4 h and reoxygenation for 2, 4, 6 h. Cell viability was detected by CCK-8 assay (*n* = 6 per group). **(B)** Ferrostatin-1 (Fer-1; 2 μM) attenuates H/R-induced cell death. Cell viability was detected by CCK-8 assay (*n* = 6 per group) **(C)** Representative fluorescent images of ROS staining by using DCFH-DA (scale bar, 100 μm), **(D)** and quantification of ROS fluorescence intensity (*n* = 5 per group). **(E–H)** RT‒qPCR results of PTGS2, ACSL4, TFRC and HMOX1 in H9C2 cells pretreated with Fer-1 and subjected to H/R (*n* = 6 per group). **(I)** Effects of DAPA administration for eight or 24 h on the survival of H9C2 cells detected by CCK-8 assay (n = 6 per group). **(J)** Effects of DAPA treatment on H/R-induced cell death detected by CCK-8 (*n* = 6 per group). **(K)** H/R-induced cell viability (%) of DAPA at different concentrations, cell viability was detected by CCK-8 assay (*n* = 6 per group). **(L)** Measurement of SOD activity by a commercial kit (*n* = 6 per group). **(M)** Total cellular LDH release was measured by a commercial kit (*n* = 4–6 per group). **(N–O)** Western blot analysis of PTGS2 in H9C2 cells after treatment with DAPA (2.5 or 5 μM) (*n* = 5 per group). Data are expressed as mean ± SD; Statistical analysis: One-way ANOVA followed by Tukey’s correction for *post hoc* multiple comparisons or Dunnett’s multiple comparison tests; #*p* < 0.05, ##*p* < 0.01 vs Control group; *p < 0.05, **p < 0.01 vs H/R group.

To investigate MIRI, we performed H/R in H9C2 cardiomyocytes. We initially performed hypoxia for 4 h followed by a time gradient of reoxygenation to measure cell activity using CCK-8. The results revealed that the cell activity of H9C2 cells decreased significantly after H/R (Figure 1A). To prove that cardiac H/R injury may be related to ferroptosis, we treated H9C2 cells with ferrostatin (Fer-1) before H/R. The results confirmed that Fer-1 improved H9C2 H/R cell activity (Figure 1B) and reduced ROS production (Figures 1C, D). In addition, in H/R-induced H9C2 cell death, treatment with Fer-1 significantly reduced the mRNA level of the molecular marker of ferroptosis and the most critical lipid peroxidase, prostaglandin endoperoxide synthase 2 (PTGS2) and acyl-CoA synthetase long-chain family member 4 (ACSL4) (Figures 1E, F). We also examined the mRNA expression of genes associated with iron metabolism in ferroptosis, such as TFRC and HMOX1, which showed similar changes (Figures 1G, H). These results suggest that ferroptosis activation may play a critical role in the pathogenesis of myocardial ischemia/reperfusion.

The results showed that DAPA had no cytotoxic effect on H9C2 cells under normal culture up to 200 μM (Figure 1I). Meanwhile, the H/R model resulted in a significant decrease in H9C2 cell viability, which was reversed by DAPA treatment in a dose-dependent manner, indicating that DAPA had a protective effect on H/R *in vitro* (Figure 1J). On the other hand, under H/R conditions, cell viability decreased further when DAPA reached a maximum of 40 μM (Figure 1K). As shown in Figure 1L, DAPA prevented the decrease in SOD activity, and LDH release in H/R-treated cells was also prevented by DAPA (Figure 1M). The protein level of PTGS2 was significantly decreased in the DAPA-treated group compared with the H/R group (Figures 1N, O). The above results indicated the protective effect of DAPA on H/R in cardiomyocytes.

### 3.2 Effects of dapagliflozin on ROS and lipid peroxidation accumulation *in Vitro*

**FIGURE 2:**
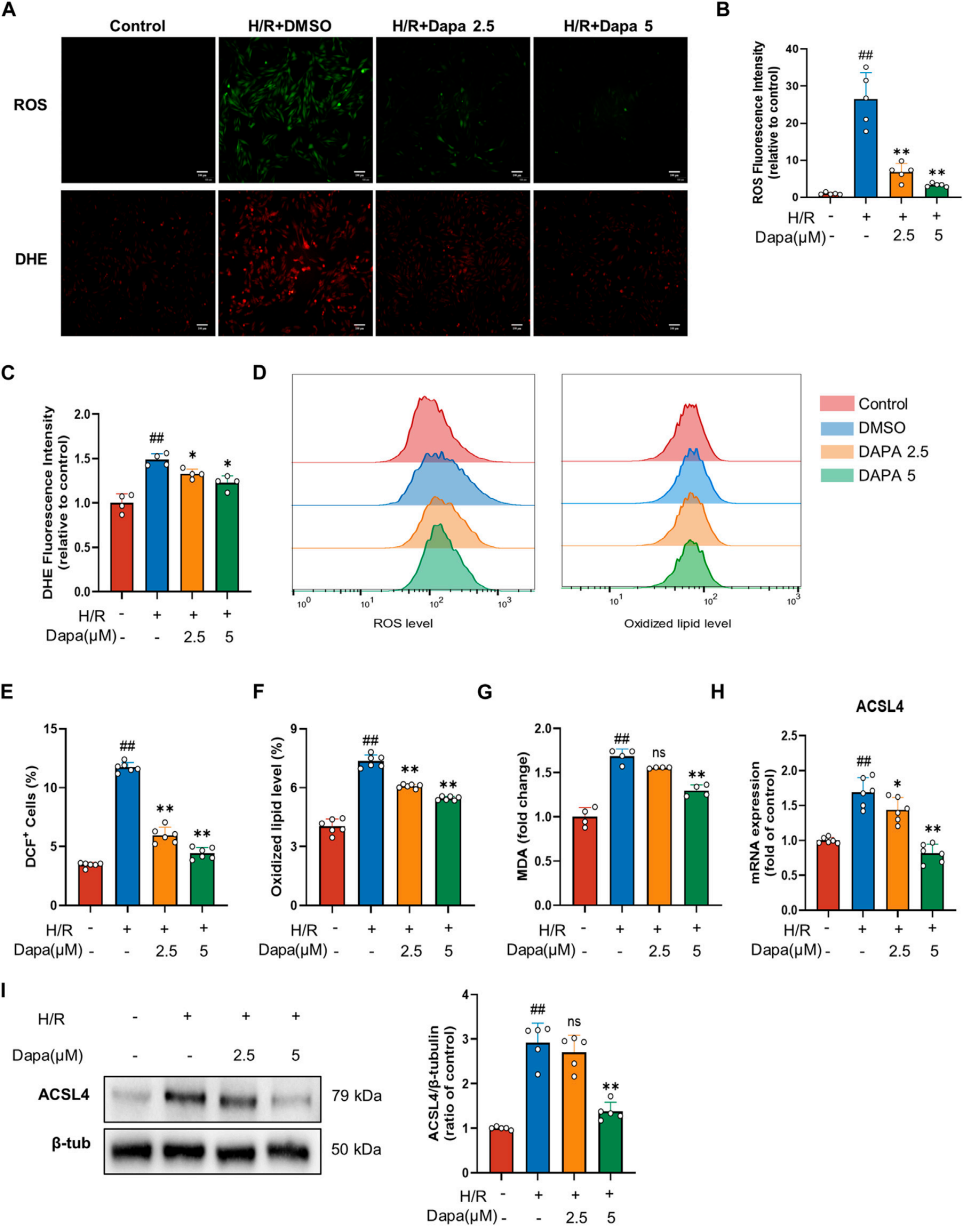
Dapagliflozin ameliorates H/R-induced ROS and lipid peroxidation in H9C2 cells. **(A–C)** Representative fluorescence images of ROS and DHE staining (scale bar, 100 μM) and quantification of fluorescence intensity (*n* = 4, 5 per group). **(D–F)** The levels of lipid ROS were captured by using 2,7-dichlorofluorescein diacetate, oxidized lipids were captured by using C11 BODIPY and measured by flow cytometry in H9C2 cells pretreated with or without DAPA (*n* = 6 per group). **(G)** MDA content was assayed by a commercial kit (*n* = 4 per group). **(H)** The mRNA level of ACSL4 was measured by qPCR (*n* = 6 per group). **(I)** The protein level was measured by western blotting after treatment with 2.5 or 5 μM DAPA (*n* = 5 per group). Data are expressed as mean ± SD; One-way ANOVA followed by Tukey’s correction for *post hoc* multiple comparisons or Dunnett’s multiple comparison tests; #*p* < 0.05, ##*p* < 0.01 vs Control group; *p < 0.05, **p < 0.01 vs H/Rgroup.

Lipid peroxidation is described as a process in which oxidants, such as ROS, attack lipids, which is a key feature of ferroptosis, so we examined the effects of DAPA on ROS production and lipid peroxidation. H/R modelcaused a distinct increase in ROS and DHE. Nevertheless, DAPA pretreatment effectively prevented-reversed these changes (Figures 2A–D). In addition, DAPA alleviated the H/R-induced increase in MDA content (Figure 2E). ACSL4 is the most critical enzyme in lipid peroxidation in ferroptosis. As expected, the mRNA (Figure 2F) and protein levels (Figures 2G, H) of ACSL4 increased significantly under H/R, which was effectively relieved in the dapagliflozin treatment groups.

### 3.3 Dapagliflozin prevented glutathione depletion in H9C2 cells

**FIGURE 3:**
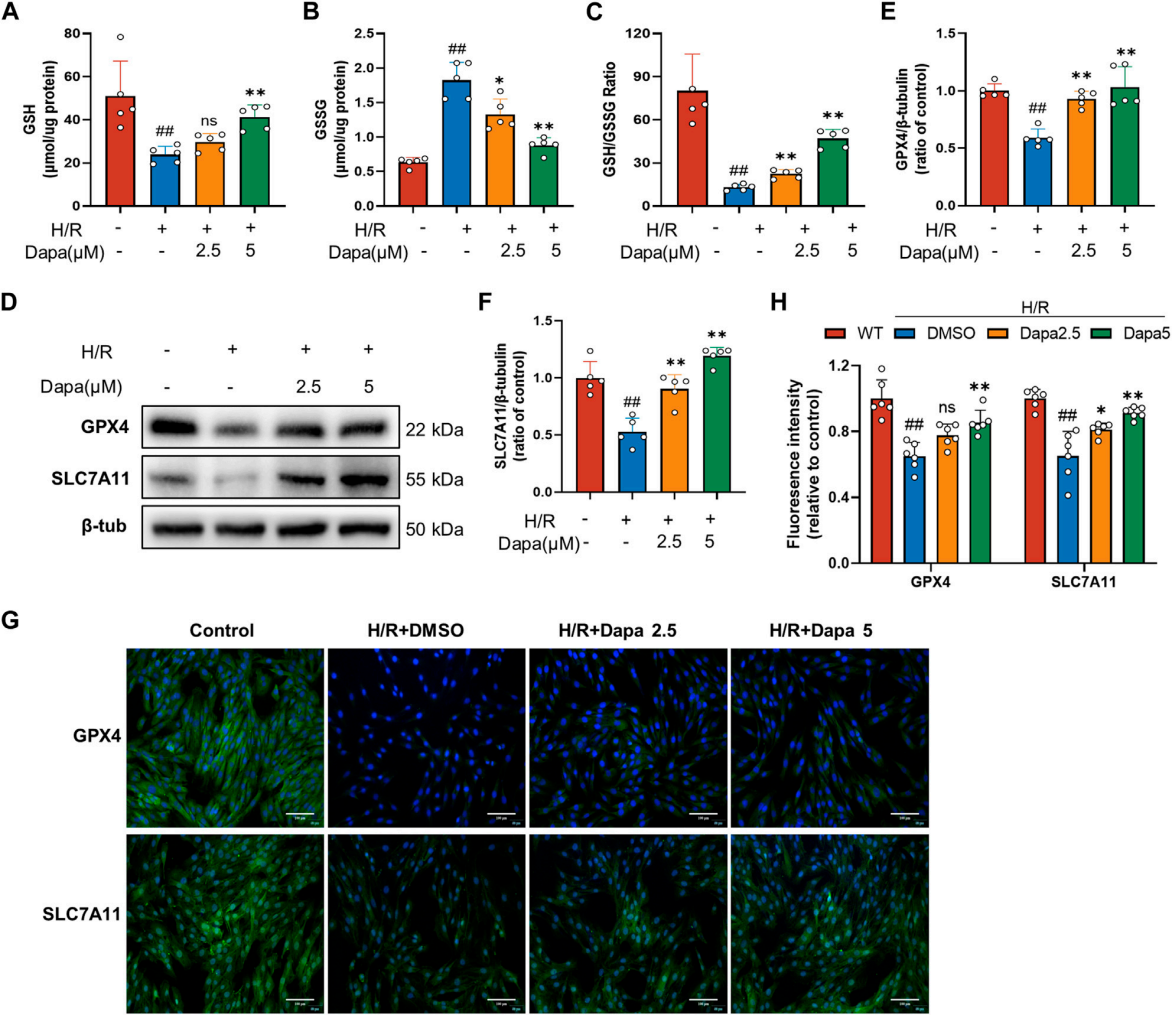
Dapagliflozin improves the glutathione antioxidant system in H/R. **(A–C)** GSH, GSSG and GSH/GSSG ratio changes in H9C2 cells assayed by a commercial kit (*n* = 5 per group). **(D–F)** The protein levels of GPX4 and SLC7A11 were measured by western blotting. Relative densitometry values are shown (*n* = 5 per group). **(G, H)** Representative immunofluorescence images of GPX4 and SLC7A11 (scale bar, 100 μm) and quantitative analyses (*n* = 6 per group).Data are expressed as mean ± SD; One-way ANOVA followed by Tukey’s correction for *post hoc* multiple comparisons or Dunnett’s multiple comparison tests; #*p* < 0.05, ##*p* < 0.01 vs Control group; *p < 0.05, **p < 0.01 vs H/R group.

Since glutathione derivation was demonstrated to be a hallmark of ferroptosis, we next explored whether dapagliflozin affected glutathione depletion. As shown in Figures 3A–C, antioxidant enzyme GSH activity was significantly reduced during H/R, which was effectively ameliorated by DAPA. The SLC7A11/GPX4 axis is recognized as the primary defense mechanism against ferroptosis with the help of GSH. The protein levels of GPX4 and SLC7A11, glutathione metabolism genes, were decreased in the H/R group compared with the control group, and DAPA pretreatment ameliorated this alteration (Figures 3D–F), which was further evaluated by immunofluorescence and quantitative analyses (Figures 3G, H).

### 3.4 Dapagliflozin regulates iron metabolism and improves mitochondrial function *in Vitro*

**FIGURE 4:**
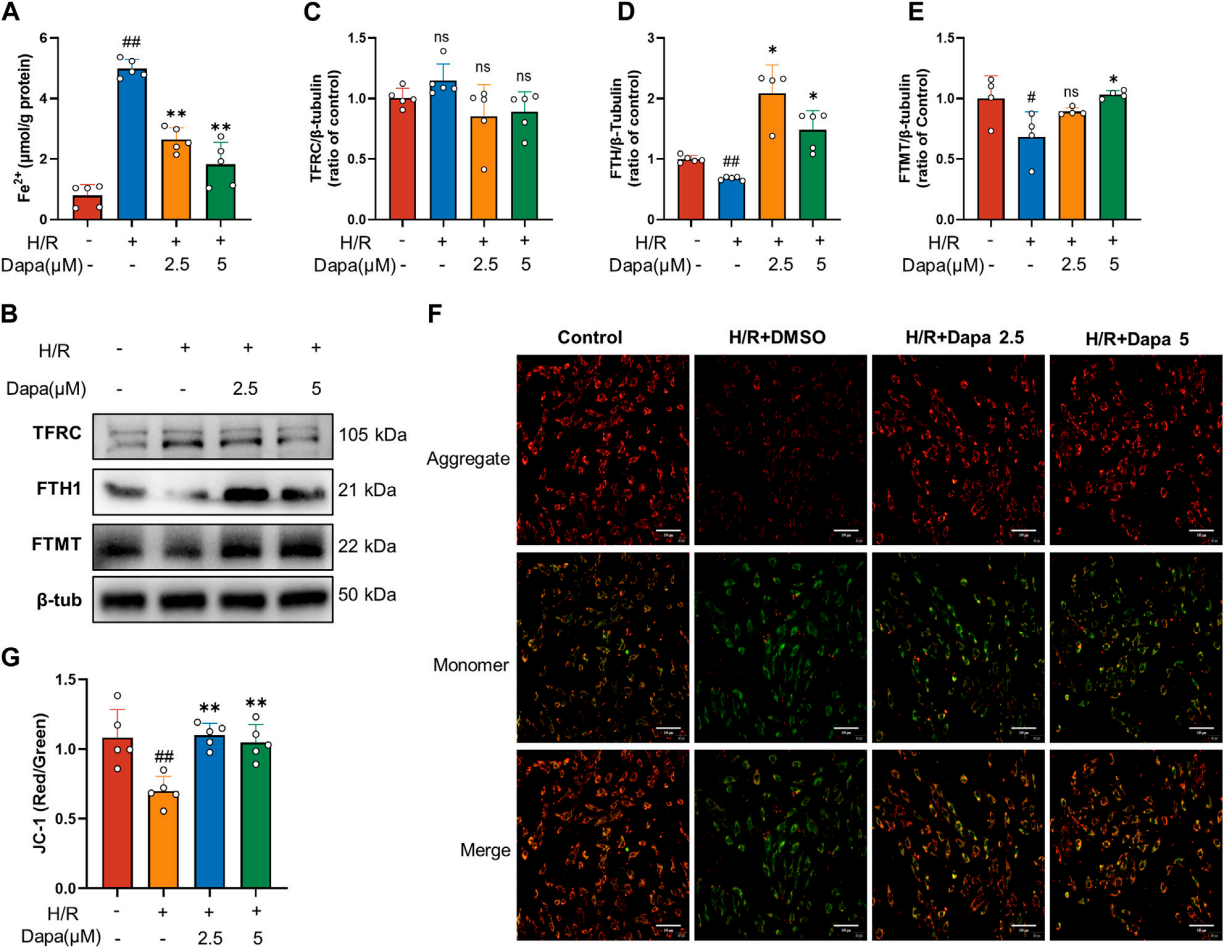
Dapagliflozin regulates intracellular iron metabolism and mitochondrial function. **(A)** Intracellular Fe^2+^ accumulation was assayed by a commercial kit (*n* = 5 per group). **(B–E)** The protein levels of TFRC, FTH1 and FTMT were measured by western blotting and quantitative analyses (*n* = 4, 5 per group). **(F, G)** Representative fluorescence images of JC-1 staining and quantitative analyses according to the red optical density/green optical density (scale bar, 100 μm, *n* = 5 per group). Data are expressed as mean ± SD; One-way ANOVA followed by Tukey’s correction for *post hoc* multiple comparisons or Dunnett’s multiple comparison tests; #*p* < 0.05, ##*p* < 0.01 vs Control group; *p < 0.05, **p < 0.01 vs H/R group.

As the central mediator of ferroptosis, iron overload produces excess ROS by the Fenton reaction, leading to mitochondrial and cell damage (Li and Zhang, 2021). We investigated whether dapagliflozin affected iron metabolic indicators and mitochondrial membrane potential. The results showed that the intracellular Fe^2+^ level was significantly increased in the H/R group, and pretreatment with dapagliflozin reduced intracellular Fe^2+^ levels (Figure 4A). To unveil the intrinsic molecular mechanism of cumulative ferrous iron, we further explored the expression of genes related to extracellular iron absorption and intracellular iron storage. Transferrin receptor 1 (TFRC), ferritin, and mitochondrial ferritin (FTMT) play important roles in iron metabolism by regulating iron uptake and storage, respectively. The level of TFRC tended to be upregulated during H/R, but the difference was not statistically significant, while H/R significantly decreased FTH and FTMT levels (Figures 4B–E). To detect mitochondrial dynamics, JC-1 staining is shown in Figure 4F. A significant decrease in red fluorescence was remarkable in the H/R group, leading to a drop in the ratio of red to green fluorescence, compared to the control group. In contrast, dapagliflozin treatment reversed these changes (Figure 4G). These findings demonstrate that dapagliflozin regulates iron metabolism and improves mitochondrial function.

### 3.5 Dapagliflozin alleviates MIRI *in Vivo*

**FIGURE 5:**
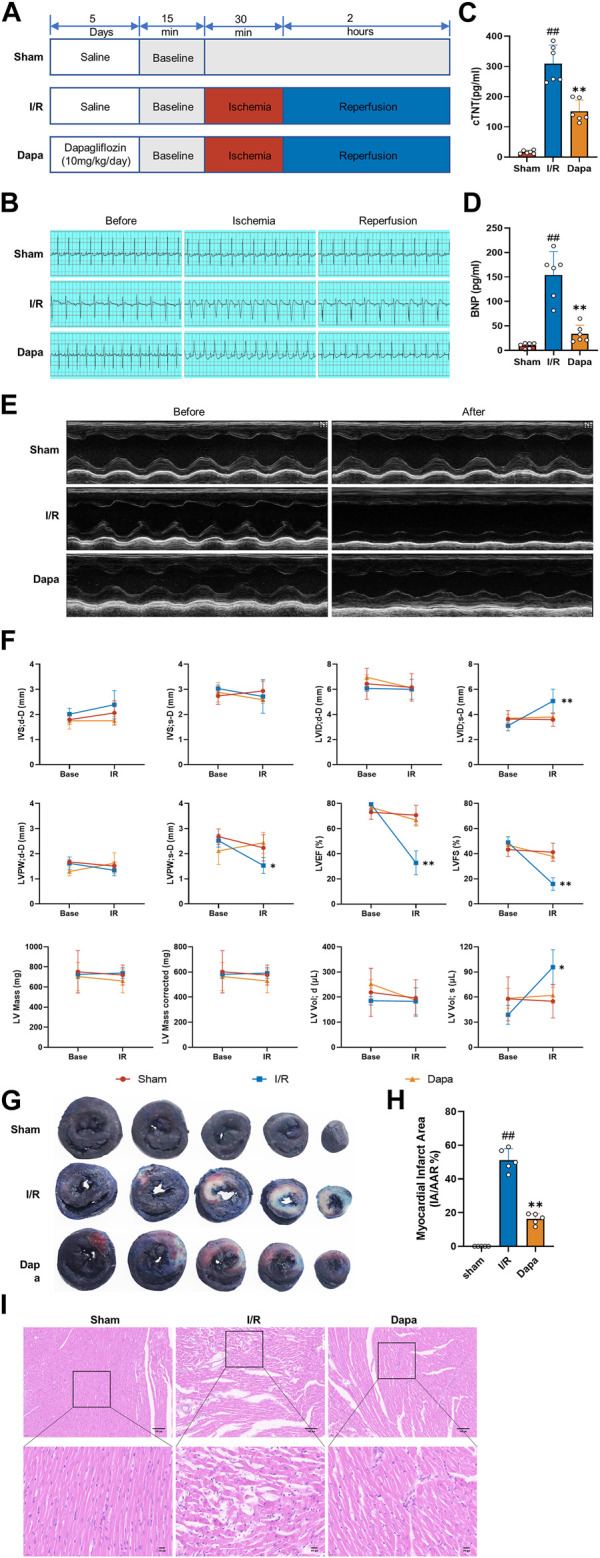
The protective effect of dapagliflozin on myocardial I/R injury *in vivo*. **(A)** Experimental protocols. **(B)** Typical ECG segments in the basic state after 30 min of ligation and reperfusion for 2 h **(C, D)** cTnT and BNP concentrations in each group were detected by ELISA kit (*n* = 6 per group). **(E)** Representative transthoracic echocardiography images of the basal state and after reperfusion. **(F)** Cardiac echocardiography parameters of the experimental rats, including IVS, LVID, LVPW, LVEF (%), LVFS(%), LV mass and volume (*n* = 5 per group). **(G)** Representative cross-section images of Evans Blue/TTC double-stained ventricle hearts subjected to I/R in the absence or the presence of Dapa. The red regions represent the area at-risk (TTC stained), the blue regions represent the non-affected areas (Evans Blue stained), and the white regions represent the infarct areas (*n* = 5 per group). **(H)** Quantitative analysis of hearts showed a significant reduction in the percentage of white/white + red areas (IA/AAR) of the hearts. **(I)** Representative images of the left ventricle stained with hematoxylin and eosin (scale bar, 100 μm). Data are expressed as mean ± SD; One-way ANOVA followed by Turkey *post hoc* tests for TnT, BNP and infarct area analysis, 2-way ANOVA for repeated measures followed by Sidak’s multiple comparison tests for echocardiography parameters analysis; #*p* < 0.05, ##*p* < 0.01 vs Sham group; *p < 0.05, **p < 0.01 vs I/R group.

To evaluate the effect of dapagliflozin on myocardial I/R injury, rats were pretreated with DAPA for 5 days before myocardial I/R surgery (Figure 5A). ST-segment elevation and reperfusion arrhythmia confirmed the success of the I/R procedure. As shown in Figure 5B, I/R stimulated the ST segment to be significantly elevated, and arrhythmias such as premature ventricular contractions or ventricular tachycardia occurred after reperfusion. The DAPA group had decreased ST segment elevation and reperfusion arrhythmia and significantly ameliorated the increased cTnT and BNP levels (Figures 5C, D). Representative echocardiographic images in M-mode showed that the DAPA group had higher LVPWs, LVFS and LVEF but lower LVIDs than the control group (Figures 5E, F). I/R exposure resulted in myocardial structural damage, disordered fiber arrangement, reduced myocardial cells, nuclear shrinkage, and inflammatory cell infiltration. In addition, Figures 5G, H shows that myocardial infarct size (%) was significantly higher in the I/R group than in the sham group, and pretreatment of DAPA decreased the infarct size. The histopathological changes were significantly attenuated in the DAPA group (Figure 5I). These results indicate that dapagliflozin reduces myocardial I/R injury and improves cardiac function in rats.

### 3.6 Dapagliflozin reduced MIRI-induced ferroptosis *in vivo*

**FIGURE 6:**
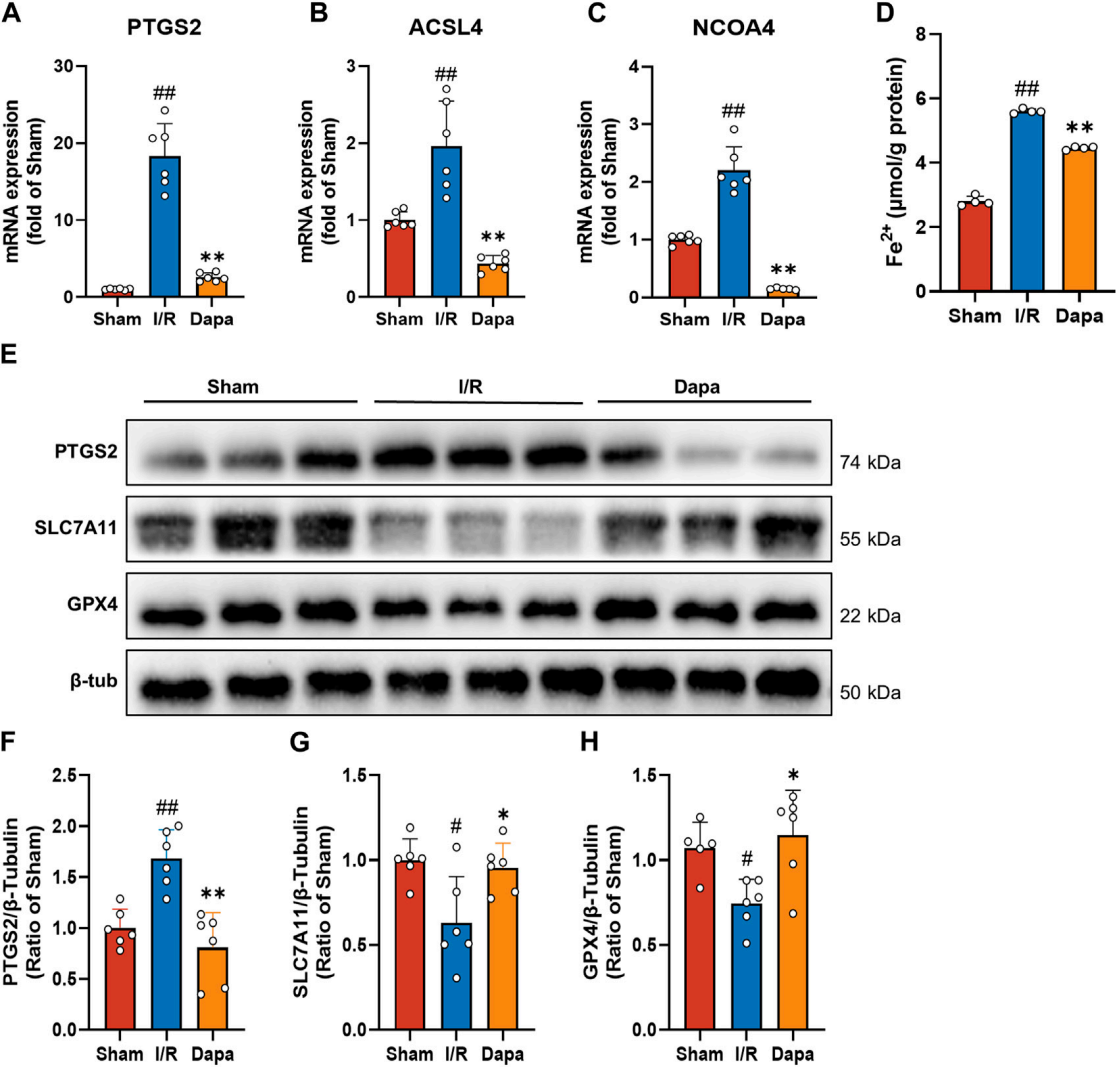
Dapagliflozin attenuates I/R-mediated ferroptosis. **(A–C)** The mRNA levels of PTGS2, ACSL4 and NCOA4 were measured by qPCR (*n* = 6 per group). **(D)** Ferrous iron levels in myocardial tissue were determined by a commercial kit (*n* = 4 per group). **(E–H)** Myocardial tissue protein levels of PTGS2, SLC7A11 and GPX4 were measured by western blotting (*n* = 6 per group).; Data are expressed as mean ± SD; One-way ANOVA followed by Tukey’s correction for *post hoc* multiple comparisons or Dunnett’s multiple comparison tests; #*p* < 0.05, ##*p* < 0.01 vs Sham group; *p < 0.05, **p < 0.01 vs I/R group.

In terms of molecular mechanisms, there was a significant increase in PTGS2, ACSL4, and NCOA4 mRNA levels in the I/R group compared to the control group, and the DAPA group exhibited reversed changes (Figures 6A–C). As depicted in Figure 6D, I/R surgery greatly facilitated the generation of Fe^2+^, and DAPA reduced intracellular ferrous iron overload. We found that PTGS2 was dramatically upregulated and SLC7A11/GPX4 protein levels were downregulated in the I/R group compared with the sham group. Non-etheless, DAPA successfully reduced PTGS2 levels and promoted SLC7A11/GPX4 axis expression (Figures 6E–H), suggesting that DAPA reduces ferroptosis in myocardial I/R rats.

### 3.7 Predicting and verifying the regulatory effect of dapagliflozin on MAPK in MIRI-induced ferroptosis

#### 3.7.1 Candidate targets of dapagliflozin and myocardial I/R injury-induced ferroptosis.

**FIGURE 7:**
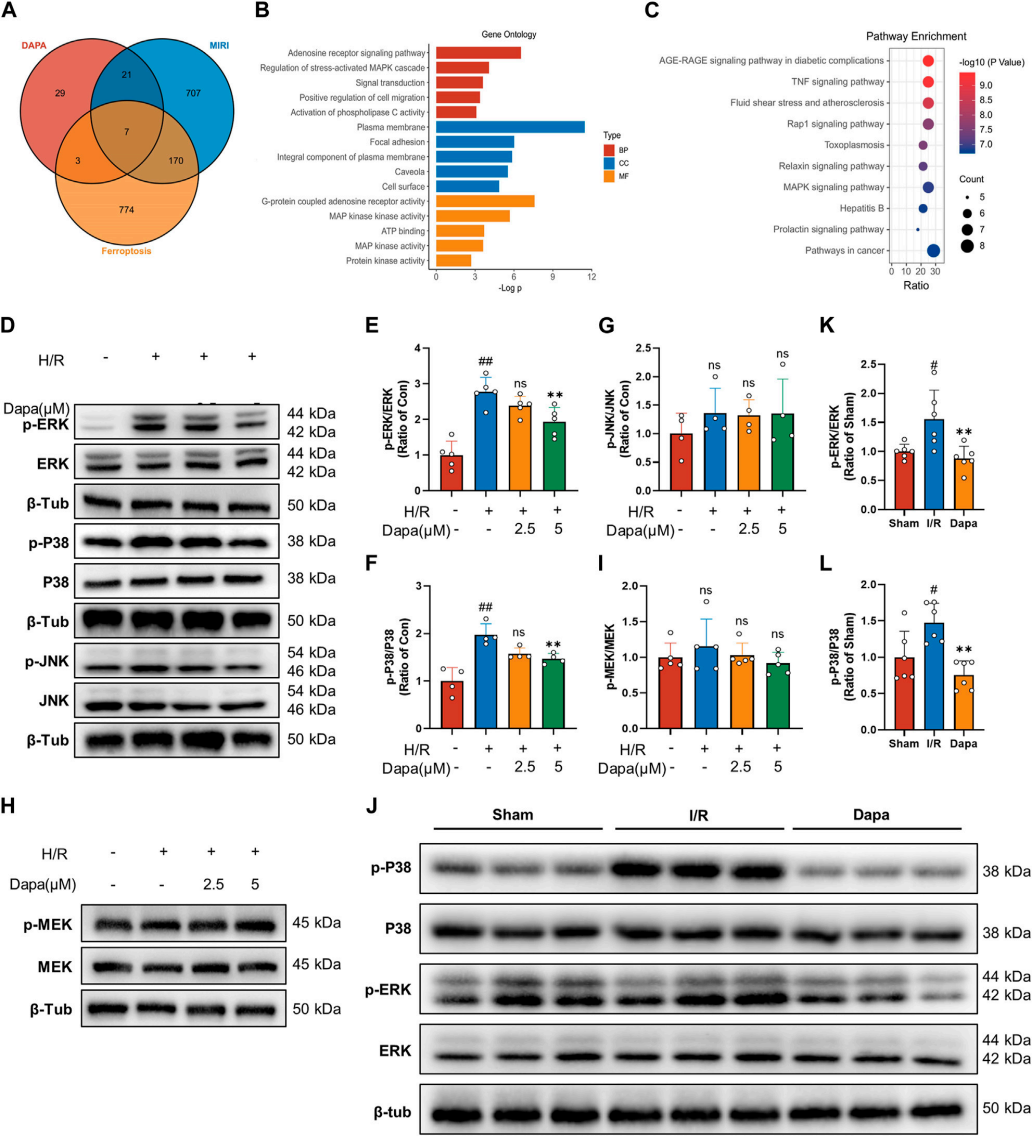
The effect of dapagliflozin on the MAPK pathway. **(A)** Venn diagram of dapagliflozin potential targets intersecting with MIRI targets and ferroptosis targets. **(B, C)** GO functional annotation and KEGG pathway analysis. **(D–G)** Total cell lysates were immunoblotted for phospho-ERK/ERK, phospho-p38 MAPK/p38 MAPK, phospho-JNK/JNK and quantitative analyses (*n* = 4, 5 per group). **(H, I)** The protein levels of phosphor-MEK/MEK were measured by western blotting *in vitro* (*n* = 5 per group). **(J–L)** Phosphorylated ERK/ERK and phosphorylated p38 MAPK/p38 MAPK *in vivo* experiments were detected by Western blotting and quantitative analyses (*n* = 6 per group). Data are expressed as mean ± SD; One-way ANOVA followed by Tukey’s correction for *post hoc* multiple comparisons or Dunnett’s multiple comparison tests; #*p* < 0.05, ##*p* < 0.01 vs Control group (*in vitro*) or Sham group (*in vivo*); *p < 0.05, **p < 0.01 vs H/R group (*in vitro*) or I/R group (*in vivo*).

We screened 60 candidate target genes of dapagliflozin based on online database prediction. The details of these predicted targets are shown in Supplementary Table S2. For myocardial I/R injury, 834 independent genes were retrieved from the OMIM database and Gene Cards database. A total of 683 genes related to ferroptosis were collected from the FerrDb and Gene Cards databases. To obtain potential targets of dapagliflozin in the treatment of I/R related to ferroptosis, we drew a Venn diagram to cross the predicted targets of dapagliflozin and I/R-related targets, and a total of 26 overlapping targets were obtained (Figure 7A) and were submitted to the STRING platform to establish a PPI network (Supplementary Figure S1A). GO and KEGG pathway enrichment analyses indicated that the MAPK signaling pathway was the most enriched (Figures 7B, C). To more accurately elucidate the mechanism of dapagliflozin in the treatment of MIRI-related ferroptosis, we used MCODE to conduct cluster analysis on the overlapping target PPI network and obtained a potential protein functional module with the best score, as shown in Supplementary Figure S1B.

#### 3.7.2 Dapagliflozin negatively regulates the MAPK pathway *in vitro* and *in vivo*.

Bioinformatics analysis showed that the MAPK pathway was the most likely regulatory pathway among these crossed genes, which may be a potential pathway mediating the protective effect of dapagliflozin. We tested this hypothesis by examining the effect of dapagliflozin on the phosphorylation of the MAPK pathway in an *in vitro* H/R model. Western blot analysis showed that H/R-induced MAPK pathway activation was reduced by dapagliflozin, including the ERK1/2, P38 but not JNK pathways (Figures 7D–G). Among them, the phosphorylation of ERK1/2 was the most obvious, and we paid special attention to its upstream MEK1/2 and found that MEK1/2 did not change during this process (Figures 7H, I). *In vivo* experiments showed that the phosphorylation of ERK and P38 MAPK was enhanced during ischemia‒reperfusion, which was alleviated by DAPA (Figures 7J–L). Taken together, these results suggest that dapagliflozin targets MAPK but not MAPKK to regulate the ferroptosis pathway and protect the myocardium against ischemia‒reperfusion injury.

## 4 Discussion

The lack of effective treatment measures for myocardial ischemia‒reperfusion injury (MIRI) remains an urgent problem to be solved worldwide. In addition, an increasing number of studies and our previous data support that ferroptosis plays an important role in MIRI (Zhao et al., 2021; Wang Z. et al., 2022). Here, combined with network pharmacological analysis, bioinformatics analysis, and *in vitro* and *in vivo* experiments, we systematically investigated the effect of dapagliflozin on ferroptosis in cardiac ischemia/reperfusion injury. Consistent with previous studies (Kajarabille and Latunde-Dada, 2019; Lillo-Moya et al., 2021), we found that adding the ferroptosis inhibitor Fer-1 to H9C2 cardiomyocytes in the H/R model improved cell viability, LDH release, and transcriptional changes in ferroptosis marker genes, indicating that inhibiting ferroptosis may become a new target for the treatment of MIRI.

Ferroptosis is a non-apoptotic cell death involving the accumulation of lipid hydroperoxides, iron overload, and glutathione reductase depletion. Therefore, selective targeting of cardiomyocyte ferroptosis is a promising strategy to ameliorate MIRI. Clinical registry studies have shown that adjunctive deferoxamine (DFO), an iron chelator, therapy after ischemic onset ameliorates oxidative stress without limiting infarct size (Chan et al., 2012). This indicates that chelating iron alone is not enough, and it may be necessary to improve the overall state of ferroptosis. Several reports have shown that drugs such as cyanidin-3-glucoside (C3G) (Shan et al., 2021) and baicalin (Fan et al., 2021) alleviate MIRI by inhibiting ferroptosis, reducing oxidative stress and Fe^2+^ content, or inhibiting lipid peroxidation. However, the common problem is that these investigational drugs have not yet been used in clinical practice. Therefore, we investigated drugs that have the potential to reduce ferroptosis to alleviate MIRI among the drugs that are being used in clinical practice.

Sodium-glucose cotransporter two inhibition (SGLT2i) has an excellent performance in clinical trials. SGLT2i are a class of hypoglycemic drugs that affect glucose reabsorption, and they have benefits in the treatment of cardiovascular diseases other than lowering blood glucose. Clinical trials have found that SGLT2 inhibitors reduce the risk of hospitalization and death due to heart failure in patients with symptomatic heart failure. The latest research shows that in patients with a recent MI, the empagliflozin treatment effectively reduced NT-proBNP while improving echocardiographic functional and structural parameters (von Lewinski et al., 2022). The actual mechanisms that produce these beneficial effects are not fully understood. Few preclinical experiments have focused on the effect of SGLT2 inhibitors on MI without diabetes, and the proposed mechanisms were lowering intracellular Na^+^ and Ca^2+^, NHE inhibition, STAT3 and AMPK activation, CamKII inhibition, reduced inflammation, oxidative stress or modulate autophagy (Andreadou et al., 2020; Yu et al., 2021; Ma et al., 2022). To the best of our knowledge, our study is the first basic research of dapagliflozin on cardiomyocytes subjected to hypoxia/reoxygenation and is the first to investigate the role of SGLT2i on ferroptosis.

In the present study, we found that DAPA protected cardiomyocytes against H/R-induced injury. *In vivo*, DAPA reduced myocardial ischemia reperfusion arrhythmia, alleviated the decline in LVEF and LVFS after IRI, and prevented LVID enlargement. A previous *in vivo* study found that acute dapagliflozin administration during cardiac I/R injury exerted cardioprotective effects by attenuating cardiac infarct size, increasing LV function and reducing arrhythmias (Lahnwong et al., 2020), but the underlying mechanisms were not further investigated. Empagliflozin eliminated myocardial vulnerability to sudden cardiac death and reduced the susceptibility to reperfusion-induced arrhythmias post I/R injury by ligation of the left main coronary artery for 5 min followed by 20 min of reperfusion (Hu et al., 2021). We further explored the mechanism underlying the cardioprotective effect and found that DAPA reduced oxidative stress and lipid peroxidation, ameliorated glutathione depletion, regulated intracellular ferrous ions and mitochondrial transmembrane potential. Mechanistically, DAPA decreased the mRNA and protein levels of PTGS2 and ACSL4 and prevented the decrease in GPX4 and SLC7A11 transcription and protein translation. In terms of regulating iron metabolism, WB results showed that DAPA decreased TFRC to reduce iron intake and increased FTH1 and FTMT to bind more free iron. Evidence has shown that FTH-deficient cardiomyocytes have reduced SLC7A11 expression, indicating that ferritin plays an important role in protecting against cardiac ferroptosis (Fang et al., 2020). The same results were also verified *in vivo*. These suggest that dapagliflozin could suppress MIRI-induced ferroptosis.

We used a network pharmacology approach to further explore the targets of DAPA and the potential mechanisms in the treatment of MIRI-mediated ferroptosis. After integrating the information from several databases, 28 targets related to dapagliflozin were identified. To better explore the mechanism of dapagliflozin in treating MIRI, 28 targets were enriched and analyzed. The biological process and module functions were mainly involved in the regulation of the MAPK cascade and MAP or MAPK kinase activity. Pathways were enriched in the AGE-RAGE signaling pathway in diabetic complications, the TNF signaling pathway, the Rap1 signaling pathway and the MAPK signaling pathway, which was consistent with previous studies in T2DM patients with heart failure (Yue et al., 2021; Mai et al., 2022). Furthermore, after crossing with the ferroptosis database, a PPI network with the best score identified the core targets, including MAPK1 and MAPK14, which are involved in the MAPK pathway.

The MAPK family includes extracellular signal-regulated kinase (ERK), P38 and c-JUN N-terminal kinase (JNK). The MAPK pathway is involved in physiological activities such as cell growth, development, differentiation and apoptosis and regulates pathological effects such as oxidative stress, and the inflammatory response affects cardiac hypertrophy and myocardial fibrosis. Many studies have confirmed that inhibition of MAPK signaling alleviates endothelial atherosclerosis (Gencer et al., 2022), cardiac fibrosis (Wu et al., 2022), cardiac hypertrophy (Luo et al., 2021) and myocardial ischemia‒reperfusion injury (Lv et al., 2020). Several studies have demonstrated that inhibition of MAPK signaling mitigates ferroptosis. In an LPS-induced ARDS model, lipocalin-2 knockdown inhibited ferroptosis by inhibiting the MAPK/ERK pathway (Wang X. et al., 2022). Cetuximab enhances RSL3-induced ferroptosis by inhibiting the Nrf2/HO-1 axis through the activation of p38 MAPK (Yang et al., 2021). It has been found that inhibition of the IRE1/JNK pathway can alleviate I/R-induced kidney injury by inhibiting ferroptosis in I/R-induced AKI mice (Liang et al., 2022). Therefore, we focused on the protein expression and phosphorylation of the MAPK pathway and found that DAPA significantly reduced IRI-induced infarct size and improved cardiac function. This protective effect was related to the reduction in ferroptosis, and the underlying mechanism was related to the reduction in ERK1/2 and p38-MAPK phosphorylation.

In summary, we report that dapagliflozin attenuates myocardial I/R injury by reducing ferroptosis through the MAPK signaling pathway. Our findings suggest that inhibition of cardiomyocyte ferroptosis may represent a viable therapeutic approach for MIRI. Moreover, *in vivo* and *in vitro* data support that dapagliflozin plays a protective role in MIRI that is partly mediated by the reduction of ferroptosis *via* the MAPK pathway. From a clinical perspective, these findings provide the basis for broadening the indications for SGLT2 inhibitors. Future clinical trials are needed to test the benefits of this treatment strategy.

## 5 Conclusion

The present study provided evidence that dapagliflozin significantly protects against non-diabetic MIRI. The underlying mechanism might be the reduction of ferroptosis *via* the MAPK pathway. These data suggest the potential clinical utility of SGLT2 inhibitors in the prevention of myocardial ischemia‒reperfusion injury.

## Data Availability

The original contributions presented in the study are included in the article/Supplementary Material, further inquiries can be directed to the corresponding authors.

## Supplementary material

SUPPLEMENTARY FIGURE S1 PPI network and potential protein functional module. **(A)** PPI network of 26 overlapping targets. **(B)** Potential protein functional module with the best score in the PPI network.

SUPPLEMENTARY TABLE S1 Primers for RT‒PCR.

SUPPLEMENTARY TABLE S2 Swiss Target Prediction predicts potential targets of dapagliflozin.
