# Supplementary figures and images for: Dapagliflozin alleviates myocardial ischemia/reperfusion injury by reducing ferroptosis via MAPK signaling inhibition
Source: Front Pharmacol. 2023 Feb 20;14:1078205. doi: 10.3389/fphar.2023.1078205 (PMC9986553; doi:10.3389/fphar.2023.1078205)

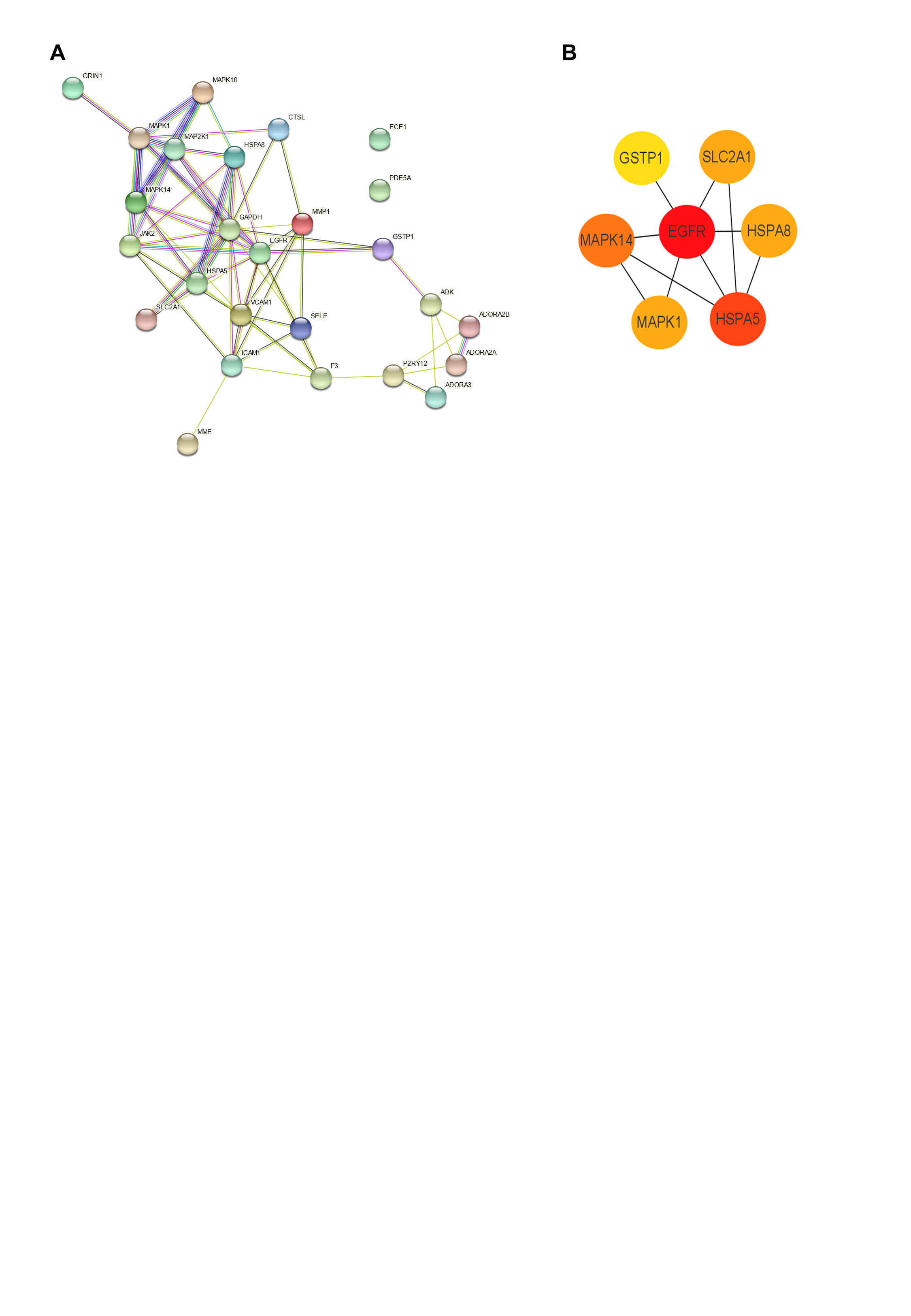

Supplement: Supplementary file 1 [file Image1.tif]
